# Trends in lifetime controlled drug use and associated risk factors among Japanese Junior High School Students: Findings from Nationwide Surveys, 2016–2024

**DOI:** 10.1002/pcn5.70241

**Published:** 2025-11-12

**Authors:** Satomi Mizuno, Satoshi Inoura, Toshihiko Matsumoto, Kunihiko Kitagaki, Akihiro Koide, Kenji Takehara, Takuya Shimane

**Affiliations:** ^1^ Department of Drug Dependence Research, National Institute of Mental Health National Center of Neurology and Psychiatry Tokyo Japan; ^2^ Department of Nursing, Faculty of Nursing Niigata Seiryo University Niigata Japan; ^3^ Social Pharmacy Laboratory, Faculty of Pharmacy Tokyo University of Pharmacy and Life Sciences Tokyo Japan; ^4^ Regulatory Science Laboratory Yokohama University of Pharmacy Kanagawa Japan; ^5^ Department of Health Policy National Centre for Child Health and Development Tokyo Japan

**Keywords:** illicit drugs, school‐based survey, social isolation, substance use attitudes, teenager

## Abstract

**Aim:**

To examine trends in the lifetime prevalence of controlled drug use among Japanese junior high school students (aged 12–15 years) between 2016 and 2024 and to identify associated sociodemographic, behavioral, and psychosocial factors.

**Methods:**

We analyzed data from 214,011 students across four nationwide surveys. The outcome was lifetime use of controlled drugs, including marijuana, solvents, methamphetamine, and new psychoactive substances. Predictors included demographic characteristics, substance‐related behaviors and attitudes, daily routines, and social relationships. Design‐weighted logistic regression was used to estimate the prevalence and assess associated risk factors.

**Results:**

The lifetime prevalence of controlled drug use showed a clear decreasing trend over the study period, declining from 0.5% (95% confidence interval [CI]: 0.4–0.5) in 2016 to 0.2% (95% CI: 0.1–0.2) in 2024. Similar downward trends were observed for all substances. While perceived drug access and permissiveness declined, tolerant attitudes toward marijuana slightly increased. Invitations to use drugs also became more common. Risk factors for controlled drug use included male sex, alcohol and tobacco use, permissive attitudes, drug invitations, irregular routines, school dissatisfaction, limited friendships, and poor family communication. The combination of multiple social risk factors, particularly when coupled with school dissatisfaction, social isolation, and limited parental consultation, significantly amplified the likelihood of drug use.

**Conclusion:**

This repeated cross‐sectional analysis indicated that lifetime use of controlled drugs among Japanese adolescents is rare and continues to decline. However, usage remains concentrated among students with overlapping behavioral and social vulnerabilities. Prevention strategies should therefore prioritize these at‐risk groups.

## Graphical abstract

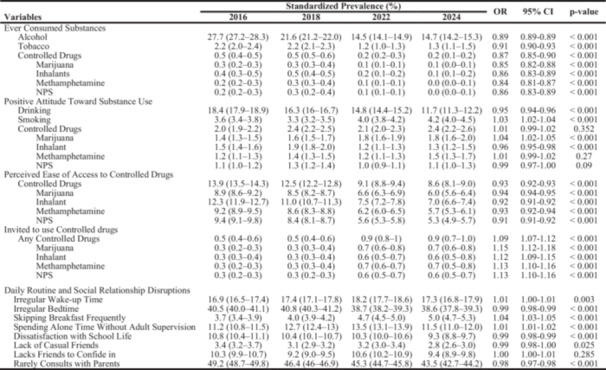

## INTRODUCTION

Adolescent use of controlled substances, including cannabis, inhalants, methamphetamine, and new psychoactive substances (NPS), is a well‐documented risk factor^1^, ^2^, ^3^, ^4^, ^5^, ^6^, ^7^, ^8^, ^9^, ^10^ for physical and mental health problems,^11^, ^12^, ^13^, ^14^, ^15^, ^16^, ^17^, ^18^, ^19^, ^20^, ^21^, ^22^, ^23^, ^24^ academic disruption,^14^, ^25^, ^26^, ^27^, ^28^ and social isolation.^12^, ^13^, ^29^, ^30^ In response to growing global concern over these risks,^31^, ^32^ many countries have implemented nationwide, school‐based surveys to monitor youth drug use. Prominent examples include the Monitoring the Future study in the United States,^33^, ^34^ the European School Survey Project on Alcohol and Other Drugs,^35^ and the Global School‐based Student Health Survey conducted by the World Health Organization.^36^, ^37^ These surveys typically report a lifetime prevalence of several percent or more among adolescents aged 13–17 years.

In contrast, countries such as South Korea, Kuwait, Iraq, Mongolia, Vietnam, Malaysia, and Japan report markedly lower prevalence rates.^38^, ^39^ In these nations, the lifetime prevalence of adolescent drug use is approximately 0.9%–1.0%, considered exceptionally low by global standards. In Japan, national surveys have consistently reported a lifetime prevalence of controlled drug use below 1% among junior high school students (ages 12–15) over the past decade.^40^ Despite its low level, this prevalence has often been regarded as a public health concern.

Although adolescent drug use in Japan remains rare, its low prevalence presents unique challenges for research and policy. Even small numerical shifts may appear exaggerated, complicating trend analysis. Moreover, most national surveys use repeated cross‐sectional designs with limited adjustment for confounders, making it difficult to determine whether the observed year‐to‐year fluctuations reflect true behavioral changes or sampling variability. Another limitation is the lack of detailed analysis of psychosocial factors, such as drug‐related attitudes, perceived accessibility, and exposure to drug offers, which are often overlooked in low‐prevalence settings. Additionally, the small number of drug‐experienced adolescents limits statistical power, impeding the detection of meaningful risk patterns. As a result, vulnerable high‐risk subgroups may go unidentified.

Japanese studies from the 1990s identified individual‐level risk factors, such as male sex, substance co‐use, and irregular routines^41^, ^42^, ^43^, ^44^, ^45^, ^46^, ^47^; however, these do not reflect recent societal changes, including the rise of social media^35^ and evolving family dynamics.^48^ International research has pointed to additional risks, such as family stress,^45^, ^49^, ^50^, ^51^, ^52^ school difficulties,^51^, ^53^, ^54^ and peer disconnection^5^, ^49^, ^50^, ^51^, ^55^, ^56^, ^57^; however, few studies have explored how these factors interact or manifest in low‐prevalence countries such as Japan.^58^, ^59^

To address these gaps, this study analyzed nationally representative data from 2016 to 2024 to estimate the standardized lifetime prevalence of controlled drug use among Japanese junior high school students and to examine temporal changes in key psychosocial factors. Using design‐weighted logistic regression, we identified sociodemographic, behavioral, and psychosocial risk factors associated with drug use. These findings aim to inform targeted prevention strategies in Japan and offer insights relevant to other low‐prevalence settings.

## METHODS

### Survey design and target population

This study analyzed data from four repeated cross‐sectional, school‐based surveys conducted biennially in 2016, 2018, 2022, and 2024. The 2020 survey was not conducted due to the coronavirus disease 2019 (COVID‐19) pandemic.

Each wave employed a self‐administered, anonymous questionnaire distributed to junior high school students aged 12–15 years from a nationally representative sample of schools.

### Stratified cluster sampling methodology

All students enrolled in randomly selected junior high schools in Japan were eligible to participate. According to the national school database used for sampling, between 3,269,341–3,486,807 students were enrolled in 10,111–10,533 public, private, and national junior high schools during each survey year.

A stratified one‐stage cluster sampling design was employed, using Japan's 47 prefectures as strata and individual schools as clusters. Within each prefecture, schools were selected using the probability‐proportional‐to‐size method based on student enrollment numbers. This approach ensured proportional representation of schools of varying sizes across all regions.

Each year, 236–244 schools were randomly selected from the national school database.

### Survey implementation procedures

Surveys were administered from September to December in each respective survey year. Prior to implementation, explanatory materials, including cooperation requests, sample questionnaires, study overviews, and response forms, were distributed to selected schools and relevant administrative bodies.

Data were collected via self‐administered paper questionnaires in 2016, 2018, and 2022. In 2024, web‐based questionnaires were also introduced at the request of some schools, in addition to the paper format.

To ensure consistency across survey years and protect student privacy, questionnaires were completed in classrooms during regular school hours (e.g., homeroom or health education). Smartphones and personal devices were not permitted; students using the online format accessed the survey only through school‐issued devices.

Teachers provided instructions based on a standardized protocol and explained the study's purpose and procedures. Students were informed that participation was voluntary and anonymous, and that they could skip any questions or withdraw at any time without penalty. Consent was obtained by checking a box at the beginning of the questionnaire. Completed paper surveys were sealed in pre‐glued opaque envelopes that could not be reopened without breaking the seal. Online responses were submitted by closing the browser window.

Data entry and initial processing were outsourced to a third‐party organization to ensure consistency and efficiency. To maintain confidentiality, school names were not collected; only anonymized school IDs were used.

### Ethical considerations

The survey protocols for all the years were approved by the Ethics Committee (A2022‐027, A2016‐022, and A2015‐128), and informed consent was obtained from all students in accordance with the Declaration of Helsinki.

### Study population

The analytical sample was defined using pre‐specified criteria. Students were excluded if their responses were largely incomplete (e.g., <50% of items answered), if they were enrolled in special education programs, or if information on their sex was missing or recorded as non‐binary. The analysis focused on students in general education programs, who comprise most Japanese adolescents and attend relatively standardized schools.

### Survey items and variables

Each survey included 32–35 items with minor variations across years. This study analyzed only the items common to all survey waves.^40^, ^60^, ^61^, ^62^, ^63^ The exact Japanese item wording is provided in a previous national report.^40^

The dependent variable was lifetime use of any controlled drug—self‐reported use of cannabis, inhalants, methamphetamine, or NPS. Although non‐medical use of over‐the‐counter (OTC) medications has become a growing concern in Japan,^57^ the survey included an OTC‐related item only in 2024 (not in 2016/2018/2022); therefore, OTC misuse was not included in the primary longitudinal analyses. Accordingly, the focus of this study was the use of regulated drugs.

Independent variables were selected based on previously published literature and theoretical relevance, and grouped into four domains: demographics, substance‐related attitudes and behaviors, lifestyle, and social relationships.^14^, ^41^, ^42^, ^43^, ^44^, ^45^, ^46^, ^47^, ^56^, ^57^, ^58^, ^59^, ^64^, ^65^, ^66^, ^67^, ^68^ Demographic variables (sex, grade, and survey year) were used as control variables. Substance‐related variables included lifetime alcohol and tobacco use, permissive attitudes toward controlled drug use, perceived ease of access to controlled drugs, and having received an invitation to use controlled drugs. Lifestyle variables included disruptions to daily routines (irregular sleep, skipping breakfast) and time without adult supervision. Based on prior Japanese research,^57^ we pre‐specified a dichotomous indicator (≥3 h vs <3 h) to proxy reduced supervision time—an “opportunity” window rather than family dysfunction—while improving model stability for rare outcomes. Social relationships were assessed with four items on peer interaction and emotional support. Social relationships were assessed using four items capturing everyday peer interaction and emotional support. These included dissatisfaction with school life, lack of casual friends to interact with, and occasionally, absence of friends to confide in, and rarely consulting parents.^57^, ^68^

### Data cleaning procedures

To ensure data quality, responses related to lifetime‐controlled drug use were cleaned using predefined consistency rules. Students were asked about their use of four specific substances—marijuana, inhalants, methamphetamine, and NPS using the following response options: (1) never used, (2) used in the past year, and (3) used more than a year ago. These responses were cross‐checked with answers to general questions on drug use. In cases of inconsistency—for example, indicating “never used” for all substances while reporting general drug use—responses were adjusted to ensure logical consistency. Approximately 0.1% of the responses were revised based on these checks.

### Statistical analyses

All statistical analyses were conducted using R (version 4.4.3) and IBM SPSS Statistics (version 29). We performed complete‐case analysis without imputation. The complex survey design accounted for stratification by prefecture, clustering by school, and post‐stratification weights based on school‐ and student‐level response rates.

We estimated both crude and standardized prevalence rates of lifetime‐controlled drug use, along with other drug‐related and psychosocial variables, for each survey year. Standardization was based on the sex and grade distribution from the 2024 survey. All analyses accounted for the complex survey design features mentioned above. Temporal trends were assessed using design‐weighted logistic regression, with survey year treated as a continuous variable.

To examine associated factors, we conducted multivariate design‐weighted logistic regression analyses using pooled data from all survey years. The outcome variable was the lifetime experience of controlled drug use (yes/no). Explanatory variables were grouped into three domains: (1) demographics, (2) attitudinal and behavioral factors, and (3) lifestyle and social relationships. Analyses were performed using the svyglm() function in R.

Three models were developed to assess the relationship between psychosocial factors and lifetime drug use. Model A included basic demographic and behavioral/attitudinal variables. Model B built upon this by adding lifestyle and social relationship variables. It also evaluated whether the combination of multiple social disconnection indicators—specifically school dissatisfaction, lack of casual friends, and infrequent parental consultation was associated with a greater risk than individual factors alone. Two‐ and three‐way interaction terms were included to assess this. Model C replaced “lack of casual friends” with “lack of friends to confide in,” to compare the impact of casual peer relationships versus emotionally supportive ones. These two friendship indicators were analyzed in separate models to avoid multicollinearity. Additionally, we conducted sensitivity analyses using simplified versions of Models B and C without interaction terms.

## RESULTS

**Figure 1:**
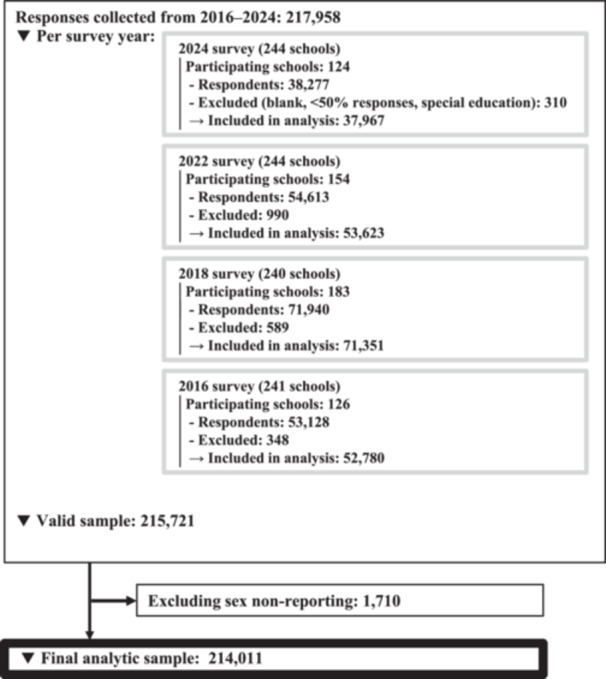
Participant Flow from National Surveys (2016–2024).

A total of 217,958 students participated in the survey between 2016 and 2024. After excluding 2,237 students with insufficient responses and 1,710 with missing or non‐binary sex data, the final analytical sample comprised 214,011 students (Figure 1). Participant characteristics are summarized in Table 1 and Table S1. The crude prevalence estimates were comparable to the standardized values, indicating minimal impact from standardization (Table S2).

**Table 1:** Basic demographic and behavioral characteristics of Junior High School Students who participated in the National Surveys (2016–2024).

	Overall *n* = 214,011		Missing value
Variable	*n*	(%)	(%)
Sex			0.0
Male	108,051	(50.5)	
Female	105,960	(49.5)	
Survey year			0.0
2016	52,681	(24.6)	
2018	71,191	(33.3)	
2022	52,670	(24.6)	
2024	37,469	(17.5)	
Grade			0.0
First (12–13‐year‐old students)	69,237	(32.4)	
Second (13–14‐year‐old students)	71,847	(33.6)	
Third (14–15‐year‐old students)	72,927	(34.1)	
Ever consumed substances			
Alcohol	41,784	(19.7)	0.9
Tobacco	3763	(1.8)	1.0
Any Controlled drugs	832	(0.4)	0.7
Marijuana	475	(0.2)	1.0
Inhalants	715	(0.3)	0.9
Methamphetamine	431	(0.2)	0.9
NPS	421	(0.2)	1.1
Positive attitude toward underage substance use			
Drinking	32,894	(15.5)	1.1
Smoking	7973	(3.8)	1.0
Any Controlled drugs	4905	(2.3)	0.8
Marijuana	3564	(1.7)	0.8
Inhalant	3361	(1.6)	0.9
Methamphetamine	2952	(1.4)	0.9
NPS	2497	(1.2)	0.9
Perceived ease of access to controlled drugs			
Any Controlled drugs	23,808	(11.3)	1.9
Marijuana	16,108	(7.7)	1.8
Inhalant	20,652	(9.8)	1.8
Methamphetamine	16,048	(7.6)	1.8
NPS	15,561	(7.4)	1.9
Invited to use controlled drugs			
Any controlled drugs	1476	(0.7)	1.6
Marijuana	1072	(0.5)	0.9
Inhalant	996	(0.5)	0.9
Methamphetamine	1016	(0.5)	1.5
NPS	955	(0.5)	1.5
Daily routine and social relationship disruptions			
Irregular or late wake‐up time	37,528	(17.6)	0.1
Irregular or late bedtime	85,323	(39.9)	0.2
Skipping breakfast frequently	8940	(4.2)	0.1
Spending alone time without adult supervision	26,253	(12.3)	0.7
Dissatisfaction with school life	22,160	(10.4)	0.4
Lack of casual friends	6570	(3.1)	0.3
Lack of friends to confide in	20,681	(9.7)	0.6
Rarely consults with parents	98,410	(46.3)	0.7

Abbreviation: NPS, new psychoactive substances.

The standardized lifetime prevalence of controlled drug use declined from 0.5% in 2016 to 0.2% in 2024 (odds ratio [OR] = 0.87), reflecting similar trends observed for alcohol and tobacco use (Table 2). The use of marijuana, inhalants, methamphetamine, and NPS also decreased. While permissive attitudes toward drug use generally declined, tolerance toward marijuana increased slightly (OR = 1.04). Perceived ease of access to drugs decreased (OR = 0.93), whereas reports of being offered drugs increased marginally (OR = 1.09). Risk‐related behaviors, such as irregular sleep, skipping breakfast, and extended unsupervised time, showed modest increases. In contrast, indicators of social disconnection school dissatisfaction, lack of casual friends, and limited communication with parents, declined gradually.

**Table 2:** Standardized prevalence and time trends in substance use, attitudes, accessibility, and daily routine and social relationship disruptions among Japanese Junior High School Students, 2016–2024.

	Standardized prevalence (%)				OR	95% CI	*p* value
Variables	2016	2018	2022	2024			
Ever consumed substances							
Alcohol	27.7 (27.2–28.3)	21.6 (21.2–22.0)	14.5 (14.1–14.9)	14.7 (14.2–15.3)	0.89	0.89–0.89	<0.001
Tobacco	2.2 (2.0–2.4)	2.2 (2.1–2.3)	1.2 (1.0–1.3)	1.3 (1.1–1.5)	0.91	0.90–0.93	<0.001
Controlled drugs	0.5 (0.4–0.5)	0.5 (0.5–0.6)	0.2 (0.2–0.3)	0.2 (0.1–0.2)	0.87	0.85–0.90	<0.001
Marijuana	0.3 (0.2–0.3)	0.3 (0.3–0.4)	0.1 (0.1–0.1)	0.1 (0.0–0.1)	0.85	0.82–0.88	<0.001
Inhalants	0.4 (0.3–0.5)	0.5 (0.4–0.5)	0.2 (0.1–0.2)	0.1 (0.1–0.2)	0.86	0.83–0.89	<0.001
Methamphetamine	0.2 (0.2–0.3)	0.3 (0.3–0.4)	0.1 (0.1–0.1)	0.0 (0.0–0.1)	0.84	0.81–0.87	<0.001
NPS	0.2 (0.2–0.3)	0.3 (0.2–0.4)	0.1 (0.1–0.1)	0.0 (0.0–0.1)	0.86	0.83–0.89	<0.001
Positive attitude toward substance use							
Drinking	18.4 (17.9–18.9)	16.3 (16–16.7)	14.8 (14.4–15.2)	11.7 (11.3–12.2)	0.95	0.94–0.96	<0.001
Smoking	3.6 (3.4–3.8)	3.3 (3.2–3.5)	4.0 (3.8–4.2)	4.2 (4.0–4.5)	1.03	1.02–1.04	<0.001
Controlled drugs	2.0 (1.9–2.2)	2.4 (2.2–2.5)	2.1 (2.0–2.3)	2.4 (2.2–2.6)	1.01	0.99–1.02	0.352
Marijuana	1.4 (1.3–1.5)	1.6 (1.5–1.7)	1.8 (1.6–1.9)	1.8 (1.6–2.0)	1.04	1.02–1.05	<0.001
Inhalant	1.5 (1.4–1.6)	1.9 (1.8–2.0)	1.2 (1.1–1.3)	1.3 (1.2–1.5)	0.96	0.95–0.98	<0.001
Methamphetamine	1.2 (1.1–1.3)	1.4 (1.3–1.5)	1.2 (1.1–1.3)	1.5 (1.3–1.7)	1.01	0.99–1.02	0.27
NPS	1.1 (1.0–1.2)	1.3 (1.2–1.4)	1.0 (0.9–1.1)	1.1 (1.0–1.3)	0.99	0.97–1.00	0.09
Perceived ease of access to controlled drugs							
Controlled drugs	13.9 (13.5–14.3)	12.5 (12.2–12.8)	9.1 (8.8–9.4)	8.6 (8.1–9.0)	0.93	0.92–0.93	<0.001
Marijuana	8.9 (8.6–9.2)	8.5 (8.2–8.7)	6.6 (6.3–6.9)	6.0 (5.6–6.4)	0.94	0.94–0.95	<0.001
Inhalant	12.3 (11.9–12.7)	11.0 (10.7–11.3)	7.5 (7.2–7.8)	7.0 (6.6–7.4)	0.92	0.91–0.92	<0.001
Methamphetamine	9.2 (8.9–9.5)	8.6 (8.3–8.8)	6.2 (6.0–6.5)	5.7 (5.3–6.1)	0.93	0.92–0.94	<0.001
NPS	9.4 (9.1–9.8)	8.4 (8.1–8.7)	5.6 (5.3–5.8)	5.3 (4.9–5.7)	0.91	0.91–0.92	<0.001
Invited to use controlled drugs							
Any controlled drugs	0.5 (0.4–0.6)	0.5 (0.4–0.6)	0.9 (0.8–1)	0.9 (0.7–1.0)	1.09	1.07–1.12	<0.001
Marijuana	0.3 (0.2–0.3)	0.3 (0.3–0.4)	0.7 (0.6–0.8)	0.7 (0.6–0.8)	1.15	1.12–1.18	<0.001
Inhalant	0.3 (0.3–0.4)	0.3 (0.3–0.4)	0.6 (0.5–0.7)	0.6 (0.5–0.8)	1.12	1.09–1.15	<0.001
Methamphetamine	0.3 (0.2–0.3)	0.3 (0.3–0.4)	0.7 (0.6–0.7)	0.7 (0.5–0.8)	1.13	1.10–1.16	<0.001
NPS	0.3 (0.2–0.3)	0.3 (0.2–0.3)	0.6 (0.5–0.7)	0.6 (0.5–0.7)	1.13	1.10–1.16	<0.001
Daily routine and social relationship disruptions							
Irregular wake‐up time	16.9 (16.5–17.4)	17.4 (17.1–17.8)	18.2 (17.7–18.6)	17.3 (16.8–17.9)	1.01	1.00–1.01	0.003
Irregular bedtime	40.5 (40.0–41.1)	40.8 (40.3–41.2)	38.7 (38.2–39.3)	38.6 (37.8–39.3)	0.99	0.98–0.99	<0.001
Skipping breakfast frequently	3.7 (3.4–3.9)	4.0 (3.9–4.2)	4.7 (4.5–5.0)	5.0 (4.7–5.3)	1.04	1.03–1.05	<0.001
Spending alone time without adult supervision	11.2 (10.8–11.5)	12.7 (12.4–13)	13.5 (13.1–13.9)	11.5 (11.0–12.0)	1.01	1.01–1.02	<0.001
Dissatisfaction with school life	10.8 (10.4–11.1)	10.4 (10.1–10.7)	10.3 (10.0–10.6)	9.3 (8.8–9.7)	0.99	0.98–0.99	<0.001
Lack of casual friends	3.4 (3.2–3.7)	3.1 (2.9–3.2)	3.2 (3.0–3.4)	2.8 (2.6–3.0)	0.99	0.98–1.00	0.025
Lacks friends to confide in	10.3 (9.9–10.7)	9.2 (9.0–9.5)	10.6 (10.2–10.9)	9.4 (8.9–9.8)	1.00	1.00–1.01	0.285
Rarely consults with parents	49.2 (48.7–49.8)	46.4 (46–46.9)	45.3 (44.7–45.8)	43.5 (42.7–44.2)	0.98	0.97–0.98	<0.001

*Note*: Prevalence estimates were directly standardized to the 2024 survey population for sex and grade.

Time trends were assessed using logistic regression models, with the survey year treated as a continuous variable and adjusted for sex and grade via post‐stratification weights.

ORs represent the direction and magnitude of change over time.

All analyses accounted for a complex survey design (sampling weights, stratification, and clustering).

Statistical significance was defined as *p* < 0.05.

Abbreviations: 95% CI, 95% confidence interval; NPS, new psychoactive substances; OR, odds ratio.

Multivariate logistic regression analysis indicated that more recent survey years (OR = 0.87) and female sex (OR = 0.77) were consistently associated with lower odds of drug use (Table 3). In Model A, significant associations were observed for lifetime alcohol use (OR = 1.40), tobacco use (OR = 3.31), permissive attitudes (OR = 7.05), perceived ease of access (OR = 4.08), and prior drug use (OR = 11.18). Model B incorporated lifestyle variables, including irregular wake‐up time (OR = 1.55), skipping breakfast (OR = 2.45), and extended unsupervised time (OR = 1.68). A notable three‐way interaction was observed between school dissatisfaction, lack of casual friends, and rare consultations with parents (OR = 9.63). Model C, which replaced “lack of confidants” instead of “lack of casual friends” with “lack of friends to confide in” yielded similar results for lifestyle variables, although interaction terms were not significant. In the simplified versions of Models B and C (excluding interaction terms), all six psychosocial variables remained significantly associated with drug use. Sensitivity analyses supported these findings, with consistent effect estimates and similar Akaike Information Criterion values across models (Table 3).

**Table 3:** Design‐weighted logistic regression models identifying factors associated with lifetime‐controlled drug use (Models A–C).

Variable	Model A		Model B		Model B simpler		Model C		Model C simpler	
	OR (95% CI)	*p* value	OR (95% CI)	*p* value	OR (95% CI)	*p*‐value	OR (95% CI)	*p*‐value	OR (95% CI)	*p* value
Survey Year	0.87 (0.84–0.91)	<0.001	0.86 (0.83–0.90)	<0.001	0.86 (0.83–0.90)	<0.001	0.86 (0.83–0.90)	<0.001	0.86 (0.83–0.90)	<0.001
Sex	0.77 (0.62–0.95)	0.014	0.56 (0.46–0.69)	<0.001	0.56 (0.46–0.69)	<0.001	0.56 (0.46–0.69)	<0.001	0.56 (0.46–0.69)	<0.001
Grade	0.97 (0.85–1.10)	0.614	1.11 (0.982–1.254)	0.095	1.11 (0.98–1.26)	0.090	1.12 (0.99–1.27)	0.067	1.12 (0.99–1.27)	0.068
Ever consumed alcohol	1.40 (1.08–1.82)	0.011								
Ever used tobacco	3.31 (2.42–4.51)	<0.001								
Positive attitude toward drinking	0.91 (0.69–1.20)	0.503								
Positive attitude toward smoking	0.90 (0.63–1.30)	0.586								
Positive attitude toward controlled drugs	7.05 (5.08–9.78)	<0.001								
Perceived ease of access to controlled drugs	4.80 (3.70–6.21)	<0.001								
Invited to use controlled drugs	11.18 (8.31–15.03)	<0.001								
Irregular wake‐up time			1.55 (1.25–1.92)	<0.001	1.55 (1.25–1.92)	<0.001	1.56 (1.26–1.93)	<0.001	1.56 (1.26–1.92)	<0.001
Irregular bedtime			1.02 (0.83–1.26)	0.854	1.01 (0.82–1.25)	0.895	1.01 (0.82–1.25)	0.919	1.01 (0.82–1.24)	0.957
Skipping breakfast frequently			2.45 (1.83–3.28)	<0.001	2.47 (1.85–3.31)	<0.001	2.46 (1.84–3.29)	<0.001	2.49 (1.86–3.32)	<0.001
Spending alone time without adult supervision			1.68 (1.36–2.07)	<0.001	1.71 (1.39–2.10)	<0.001	1.71 (1.39–2.10)	<0.001	1.73 (1.40–2.12)	<0.001
Dissatisfaction with school life			1.16 (0.69–1.94)	0.581	1.47 (1.17–1.86)	0.001	1.20 (0.69–2.07)	0.524	1.54 (1.21–1.95)	<0.001
Lack of casual friends			1.81 (0.81–4.05)	0.152	2.39 (1.84–3.11)	<0.001				
Lack of friends to confide In							1.07 (0.60–1.89)	0.823	1.59 (1.25–2.01)	<0.001
Rarely consults with parents			1.29 (1.03–1.61)	0.025	1.39 (1.14–1.70)	0.001	1.20 (0.95–1.51)	0.128	1.36 (1.11–1.66)	0.003
Dissatisfaction with School Life * Lack of casual friends			0.26 (0.05–1.41)	0.117						
Dissatisfaction with School Life * Lack of Friends to confide in							0.51 (0.17–1.56)	0.235		
Dissatisfaction with School Life * Rarely consults with parents			1.21 (0.66–2.21)	0.531			1.35 (0.71–2.58)	0.364		
Lack of casual friends * Rarely consults with parents			0.85 (0.32–2.26)	0.737						
Lack of friends to confide in * Rarely consults with parents							1.58 (0.77–3.24)	0.212		
Dissatisfaction with School Life *Lack of casual friends *Rarely consults with parents			9.63 (1.60–57.93)	0.014						
Dissatisfaction with School Life *Lack of Friends to confide in *Rarely consults with parents							2.31 (0.66–8.11)	0.190		

*Note*: Odds ratios, 95% confidence intervals, and p‐values were estimated using design‐weighted logistic regression models (svyglm), accounting for survey weights, stratification, and clustering.

In logistic regression analysis, sex was coded as 1 = *male* and 2 = *female*. Therefore, an OR less than 1 for the variable “Female” indicates that females were less likely than males to report lifetime illicit drug use.

Model A includes demographic and substance‐related variables.

Model B included lifestyle factors and indicators of social isolation with interaction terms among school dissatisfaction, lack of casual friends, and infrequent parental consultations.

Model C replaced “lack of casual friends” in Model B with “lack of friends to confide in.”

For model fit and sensitivity analysis, simplified versions of Models B and C were tested by excluding all interaction terms.

The AIC values were Model A 7239.92, Model B = 9029.92, Model C = 9056.83, simplified Model B = 9043.73, and simplified Model C = 9062.17.

Abbreviations: 95% CI, 95% confidence interval; NPS, new psychoactive substances; OR, odds ratio.

## DISCUSSION

Between 2016 and 2024, despite increases in tolerant attitudes toward cannabis and experiences of being offered drugs, the lifetime prevalence of controlled drug use among Japanese adolescents declined, reaching 0.2% in 2024. Key risk factors included permissive attitudes, perceived ease of access, lifestyle disruptions, and social isolation, particularly when multiple risk factors overlapped.

During the study period, the lifetime prevalence of controlled drug use among Japanese adolescents aged 12–15 years declined from 0.5% to 0.2%. This trend was consistent across all major substances and remained robust after adjusting for grade and sex. Over the same period, permissive attitudes toward drug use generally declined, although tolerance toward cannabis increased slightly. The proportion of students who perceived drugs as easily accessible decreased, whereas reports of being offered drugs showed a modest increase.

Even though the risk of being offered drugs increased,^14^, ^41^, ^42^, ^43^, ^44^, ^45^, ^46^, ^47^, ^56^, ^58^, ^59^, ^64^, ^65^, ^66^, ^67^, ^68^ actual drug use continued to decline. This seemingly paradoxical trend may reflect the growing influence of protective factors, such as strong internalized norms against drug use, increased health awareness, and early preventive education. The low prevalence may be attributed to a combination of strict drug control laws (e.g., criminalization of drug use), sustained school‐based prevention programs, societal disapproval, and pervasive social stigma, all of which likely act as deterrents to adolescent drug use.^57^, ^69^, ^70^, ^71^ In other words, even when adolescents are exposed to interpersonal risks, they may resist drug use due to reinforced personal values and strong societal norms. In our survey, more than 97% of students expressed opposition to drug use (Table 2), suggesting that abstinence may reflect not only behavioral restraint but also deeply rooted personal convictions and socially reinforced norms. It is also plausible that reduced peer interaction during the COVID‐19 pandemic contributed to the sharp decline observed in 2024, although this remains speculative.

While overall exposure to drugs appears to be declining, several concerns warrant attention. The slight increase in reports of drug offers suggests that some adolescents remain in high‐risk social environments. Simultaneously, the modest rise in cannabis tolerance may be influenced by global legalization trends and increased online exposure to cannabis‐related content.^16^, ^24^, ^67^, ^72^, ^73^, ^74^ These findings highlight that despite general improvements, specific subgroups may continue to face elevated risks due to their social context and evolving perceptions of certain substances.

Additionally, minor increases were observed in lifestyle disruptions, such as irregular sleep, skipping breakfast, and spending unsupervised time, all of which were significantly associated with drug use in this and prior studies.^36^, ^75^, ^76^, ^77^, ^78^, ^79^ These behavioral changes may reflect broader societal shifts, including the rise in dual‐income and single‐parent households,^48^ potentially resulting in reduced parental oversight.

Drug use among adolescents was associated with male sex, prior alcohol or tobacco use, permissive attitudes toward drug use, perceived ease of access, and experiences of being offered drugs. Lifestyle disruptions and weak connections with family and school were also identified as key risk factors. These findings align with those of previous studies conducted in Japan and internationally,^14^, ^41^, ^42^, ^43^, ^44^, ^45^, ^46^, ^47^, ^56^, ^58^, ^59^, ^64^, ^65^, ^66^, ^67^, ^68^ suggesting that adolescent drug use may be driven more by psychosocial vulnerabilities than by individual choice alone.

This study highlights the importance of weak family and school connections as significant contributors to adolescent drug use. Consistent with earlier findings, being offered drugs was strongly associated with actual use. While past research has emphasized the role of delinquent peers in promoting drug use,^49^, ^50^, ^80^, ^81^ the present findings—emphasizing both social isolation and drug offers—may appear somewhat inconsistent with the existing literature.

Several factors may help explain this discrepancy. In addition to the conventional pressure exerted by delinquent peer groups, socially isolated adolescents may be drawn to deviant groups as a means of seeking emotional connection or belonging.^82^, ^83^ Prior studies have shown that involvement in such groups can offer a sense of acceptance or a place to “fit in.”^51^, ^55^, ^84^ Moreover, in contexts where adolescents lack supportive family or social relationships, psychological vulnerabilities, such as low self‐esteem or anxiety, may play a role. Under such conditions, being offered drugs may not only present a behavioral opportunity but also be perceived as a temporary source of comfort or escape.

This study observed that the co‐occurrence of three social disconnection factors—school dissatisfaction, lack of casual friends, and limited communication with parents—was associated with a substantially higher likelihood of drug use. Specifically, the odds ratio for students experiencing all three conditions was 9.63. Table S3 indicates that the prevalence of drug use among this subgroup was 3.34%, compared to 0.25% among students without any of these risk factors a more than tenfold difference. These findings suggest that multiple forms of social isolation may have a cumulative impact on vulnerability to drug use. Furthermore, the results from Model B and its simplified version indicated that having at least one positive connection with school, peers, or parents was associated with lower odds of drug use. It is important to note, however, that the number of students reporting all three disconnection factors was relatively small (*n* = 68), and the 95% confidence interval for the corresponding interaction term was wide (1.60–57.93). This limits the precision of the OR estimate; thus, the findings should be interpreted with caution. Nonetheless, identifying such high‐risk subgroups remains important from a public health perspective, underscoring the value of targeted interventions for adolescents facing multiple psychosocial vulnerabilities.

Furthermore, the findings from Models B and C suggest that both emotionally close relationships and relatively casual social ties may offer protective effects. These observations underscore the importance of promoting diverse opportunities for social engagement as a strategy to prevent adolescent drug use.

Between 2016 and 2024, improvements were observed in school satisfaction, peer relationships, and parent–child communication among Japanese adolescents. Despite these positive trends, difficulties in interpersonal relationships remained significantly associated with drug use. This suggests that enhancements in the overall social environment may not be sufficient to protect all adolescents, particularly those experiencing persistent social disconnection. One possible explanation is that such individuals may not fully engage with or benefit from existing support systems if these are perceived as emotionally unsafe, inaccessible, or untrustworthy. In this study, students who rarely or never consulted their parents or friends were significantly more likely to report drug use. This suggests that at‐risk adolescents may be less inclined or less able to seek help or initiate communication during times of difficulty. Although these perceptions were not directly assessed in this study, previous research has emphasized the importance of adolescents' subjective experiences in shaping the effectiveness of school‐ and family‐based interventions.^85^, ^86^, ^87^ Taken together, these findings imply that preventive strategies should not only address peer norms and provide structural support but also foster a sense of belonging and emotional safety within school and family environments.^58^, ^85^, ^88^, ^89^ Moreover, the provision of alternative drug‐free environments, such as community‐based settings that facilitate non‐judgmental peer interactions, may offer critical support for socially isolated adolescents and reduce their susceptibility to drug use.

This study was a design‐based cross‐sectional analysis; therefore, its conclusions are limited to individual‐level associations. Even with that caveat, patterns observed in Japan may be informative for other low‐prevalence settings (e.g., South Korea and parts of the Middle East).^38^, ^39^ In such contexts, stringent drug laws, strong social disapproval, and internalized anti‐drug norms may contribute to lower prevalence.^90^ In Japan, conservative cultural norms alongside institutional prevention efforts may also deter initiation.^91^ By contrast, higher‐prevalence countries (e.g., the United States and parts of Europe) often report greater adolescent use in tandem with sensation‐seeking, peer normalization, and more permissive attitudes toward cannabis, trends shaped in part by legalization and media narratives that may weaken traditional deterrents.^92^, ^93^, ^94^

At the same time, our findings suggest that structural protections are not sufficient for all adolescents. Youth with weak connections to family, school, or peers, particularly those who rarely communicate with their parents or report low school satisfaction, are more likely to report drug use. Similar patterns have been observed in the United Arab Emirates, where socially isolated adolescents face increased risks despite strong legal and cultural controls.^95^ These findings underscore the need to complement legal frameworks with interventions that foster emotional connection and support.^13^, ^51^, ^85^, ^96^, ^97^, ^98^, ^99^

Overall, the findings suggest that strategies in low‐prevalence countries should not only focus on maintaining structural and legal deterrents but also prioritize the betterment of adolescents' emotional well‐being and sense of connectedness within family and school environments. Complementary measures, such as the provision of community‐based, drug‐free settings where adolescents can form meaningful relationships, may support those less likely to be reached through traditional prevention programs.

Finally, as this is a cross‐sectional study, causal inference is not possible, and structural factors were not directly measured. Future research should test these hypotheses using designs that incorporate contextual indicators (e.g., enforcement, availability, socioeconomic conditions).

This study has some limitations. All data were self‐reported and may be subject to recall or social desirability bias. Key confounders, such as parental substance use, mental health status, and family background, were not assessed. The cross‐sectional design precludes causal interpretation, and some subgroup analyses may have lacked statistical power. Selection bias is also possible, as non‐participating students may differ systematically from those included in the sample. Finally, the 2024 wave was administered in mixed modes (paper and a secure web platform). Potential response‐mode effects on self‐reports cannot be fully ruled out.

Although this study focused on illicit drugs, the increasing misuse of legal substances among Japanese adolescents, such as over‐the‐counter and prescription medications, has become a growing concern with increasing rates of overdose and addiction.^57^, ^100^, ^101^ These trends, observed in other countries as well,^102^, ^103^, ^104^ highlight the need for broader surveillance systems that capture both legal and illegal substance use to protect at‐risk youth.

Controlled drug use declined among Japanese junior high school students between 2016 and 2024, but remained concentrated among those with disrupted daily routines and indicators of social isolation. These findings underscore the need for targeted prevention interventions, even in low‐prevalence settings.

## AUTHOR CONTRIBUTIONS

Takuya Shimane, Kunihiko Kitagaki, Akihiro Koide, and Satoshi Inoura designed the preliminary experiments and established the participant database. Takuya Shimane, Kunihiko Kitagaki, Akihiro Koide, and Satoshi Inoura recruited the participants and collected the data. Takuya Shimane secured funding. Takuya Shimane and Satoshi Inoura designed the study and performed the statistical analyses. Satomi Mizuno drafted the manuscript. Takuya Shimane, Satoshi Inoura, Toshihiko Matsumoto, Kunihiko Kitagaki, Akihiro Koide, and Kenji Takehara supervised manuscript preparation. All authors have revised, reviewed, revised, and approved the final version of the manuscript for publication.

## CONFLICT OF INTEREST STATEMENT

The authors declare no conflicts of interest.

## ETHICS APPROVAL STATEMENT

This study was approved by the Ethics Committee of the National Centre of Neurology and Psychiatry, Japan (approval number: A2022‐027, A2016‐022, and A2015‐128). This study was conducted in accordance with the principles of the Declaration of Helsinki and the STROBE reporting guidelines.

## PATIENT CONSENT STATEMENT

Participants were informed of the purpose of the study, and those who expressed interest were invited to participate after providing written informed consent. Written informed consent was obtained from all study participants in both original studies.

## CLINICAL TRIAL REGISTRATION

This observational study did not qualify as a clinical trial according to the guidelines established by the International Committee of Medical Journal Editors.

## Supporting information

Supporting information 20250825.

## Data Availability

Owing to ethical and legal restrictions, the data used in this study are not publicly available.
