# Supplementary material for: Trends in lifetime controlled drug use and associated risk factors among Japanese Junior High School Students: Findings from Nationwide Surveys, 2016–2024
Source: PCN Rep. 2025 Nov 12;4(4):e70241. doi: 10.1002/pcn5.70241 (PMC12606039; doi:10.1002/pcn5.70241)
Supplement: Supplementary file 1 — Supporting information 20250825. [file PCN5-4-e70241-s001.docx]

**Supporting information**

**Supplementary Table 1. Regional Distribution of Basic Demographic Characteristics of Junior High School Students Participating in the National Surveys (2016–2024)**

|  | **Overall n = 214,011** | | **Missing Value** |
| --- | --- | --- | --- |
| **Variable** | **n** | **(%)** | **(%)** |
| Participants in each prefecture |  |  | 0.0 |
| Hokkaido | 5,959 | (2.8) |  |
| Aomori | 2,108 | (1.0) |  |
| Iwate | 2,325 | (1.1) |  |
| Miyagi | 3,355 | (1.6) |  |
| Akita | 281 | (0.1) |  |
| Yamagata | 1,426 | (0.7) |  |
| Fukushima | 2,190 | (1.0) |  |
| Ibaraki | 3,968 | (1.9) |  |
| Tochigi | 4,835 | (2.3) |  |
| Gunma | 2,647 | (1.2) |  |
| Saitama | 13,347 | (6.2) |  |
| Chiba | 10,578 | (4.9) |  |
| Tokyo | 19,312 | (9.0) |  |
| Kanagawa | 11,393 | (5.3) |  |
| Niigata | 2,673 | (1.2) |  |
| Toyama | 1,976 | (0.9) |  |
| Ishikawa | 2,647 | (1.2) |  |
| Fukui | 1,810 | (0.8) |  |
| Yamanashi | 1,181 | (0.6) |  |
| Nagano | 3,781 | (1.8) |  |
| Gifu | 3,105 | (1.5) |  |
| Shizuoka | 6,251 | (2.9) |  |
| Aichi | 15,476 | (7.2) |  |
| Mie | 3,797 | (1.8) |  |
| Shiga | 2,670 | (1.2) |  |
| Kyoto | 3,149 | (1.5) |  |
| Osaka | 12,931 | (6.0) |  |
| Hyogo | 10,061 | (4.7) |  |
| Nara | 2,076 | (1.0) |  |
| Wakayama | 1,461 | (0.7) |  |
| Tottori | 1,289 | (0.6) |  |
| Shimane | 904 | (0.4) |  |
| Okayama | 2,105 | (1.0) |  |
| Hiroshima | 5,652 | (2.6) |  |
| Yamaguchi | 3,839 | (1.8) |  |
| Tokushima | 2,263 | (1.1) |  |
| Kagawa | 2,926 | (1.4) |  |
| Ehime | 1,871 | (0.9) |  |
| Kochi | 1,230 | (0.6) |  |
| Fukuoka | 7,641 | (3.6) |  |
| Saga | 1,082 | (0.5) |  |
| Nagasaki | 2,626 | (1.2) |  |
| Kumamoto | 4,257 | (2.0) |  |
| Oita | 3,107 | (1.5) |  |
| Miyazaki | 3,204 | (1.5) |  |
| Kagoshima | 4,301 | (2.0) |  |
| Okinawa | 6,945 | (3.2) |  |

**Supplementary Table 2. Crude and Standardized Prevalence Estimate (2016–2024)**

| **Variables** |  | **2016** | **2018** | **2022** | **2024** |
| --- | --- | --- | --- | --- | --- |
| Ever Consumed Substances |  |  |  |  |  |
| Alcohol | Crude (95% CI) | 27.8 (27.3–28.4) | 21.9 (21.5–22.2) | 14.6 (14.2–15) | 14.7 (14.2–15.2) |
|  | Standardized (95% CI) | 27.7 (27.2–28.3) | 21.6 (21.2–22) | 14.5 (14.1–14.9) | 14.7 (14.2–15.3) |
| Tobacco | Crude (95% CI) | 2.2 (2–2.4) | 2.2 (2.1–2.4) | 1.2 (1.1–1.3) | 1.3 (1.1–1.5) |
|  | Standardized (95% CI) | 2.2 (2–2.4) | 2.2 (2.1–2.3) | 1.2 (1–1.3) | 1.3 (1.1–1.5) |
| Any Controlled Drugs | Crude (95% CI) | 0.5 (0.4–0.5) | 0.5 (0.5–0.6) | 0.2 (0.2–0.3) | 0.2 (0.1–0.2) |
|  | Standardized (95% CI) | 0.5 (0.4–0.5) | 0.5 (0.5–0.6) | 0.2 (0.2–0.3) | 0.2 (0.1–0.2) |
| Marijuana | Crude (95% CI) | 0.3 (0.2–0.3) | 0.3 (0.3–0.4) | 0.1 (0.1–0.1) | 0.1 (0–0.1) |
|  | Standardized (95% CI) | 0.3 (0.2–0.3) | 0.3 (0.3–0.4) | 0.1 (0.1–0.1) | 0.1 (0–0.1) |
| Inhalants | Crude (95% CI) | 0.4 (0.3–0.5) | 0.5 (0.4–0.5) | 0.2 (0.1–0.2) | 0.1 (0.1–0.2) |
|  | Standardized (95% CI) | 0.4 (0.3–0.5) | 0.5 (0.4–0.5) | 0.2 (0.1–0.2) | 0.1 (0.1–0.2) |
| Methamphetamine | Crude (95% CI) | 0.2 (0.2–0.3) | 0.3 (0.3–0.4) | 0.1 (0.1–0.1) | 0 (0–0.1) |
|  | Standardized (95% CI) | 0.2 (0.2–0.3) | 0.3 (0.3–0.4) | 0.1 (0.1–0.1) | 0.0 (0–0.1) |
| NPS | Crude (95% CI) | 0.2 (0.2–0.3) | 0.3 (0.3–0.4) | 0.1 (0.1–0.1) | 0.0 (0–0.1) |
|  | Standardized (95% CI) | 0.2 (0.2–0.3) | 0.3 (0.2–0.4) | 0.1 (0.1–0.1) | 0.0 (0–0.1) |
| Positive Attitude Toward Underage Substance Use |  |  |  |  |  |
| Alcohol | Crude (95%CI) | 18.4 (17.9–18.9) | 16.5 (16.2–16.9) | 14.8 (14.4–15.3) | 11.7 (11.3–12.2) |
|  | Standardized (95%CI) | 18.4 (17.9–18.9) | 16.3 (16–16.7) | 14.8 (14.4–15.2) | 11.7 (11.3–12.2) |
| Tobacco | Crude (95% CI) | 3.6 (3.4–3.8) | 3.4 (3.2–3.6) | 4.0 (3.8–4.2) | 4.2 (3.9–4.5) |
|  | Standardized (95% CI) | 3.6 (3.4–3.8) | 3.3 (3.2–3.5) | 4.0 (3.8–4.2) | 4.2 (4–4.5) |
| Any Controlled Drugs | Crude (95% CI) | 2 (1.9–2.2) | 2.4 (2.2–2.5) | 2.1 (2–2.3) | 2.4 (2.2–2.6) |
|  | Standardized (95% CI) | 2 (1.9–2.2) | 2.4 (2.2–2.5) | 2.1 (2–2.3) | 2.4 (2.2–2.6) |
| Marijuana | Crude (95% CI) | 1.4 (1.3–1.5) | 1.6 (1.5–1.7) | 1.8 (1.6–1.9) | 1.8 (1.6–2) |
|  | Standardized (95% CI) | 1.4 (1.3–1.5) | 1.6 (1.5–1.7) | 1.8 (1.6–1.9) | 1.8 (1.6–2) |
| Inhalants | Crude (95% CI) | 1.5 (1.4–1.6) | 1.9 (1.8–2) | 1.2 (1.1–1.3) | 1.3 (1.2–1.5) |
|  | Standardized (95% CI) | 1.5 (1.4–1.6) | 1.9 (1.8–2) | 1.2 (1.1–1.3) | 1.3 (1.2–1.5) |
| Methamphetamine | Crude (95% CI) | 1.2 (1.1–1.3) | 1.4 (1.3–1.5) | 1.2 (1.1–1.3) | 1.5 (1.3–1.7) |
|  | Standardized (95% CI) | 1.2 (1.1–1.3) | 1.4 (1.3–1.5) | 1.2 (1.1–1.3) | 1.5 (1.3–1.7) |
| NPS | Crude (95% CI) | 1.1 (1–1.2) | 1.3 (1.2–1.4) | 1 (0.9–1.1) | 1.1 (1–1.3) |
|  | Standardized (95% CI) | 1.1 (1–1.2) | 1.3 (1.2–1.4) | 1 (0.9–1.1) | 1.1 (1–1.3) |
| Perceived Ease of Access to Controlled Drugs |  |  |  |  |  |
| Any Controlled Drugs | Crude (95% CI) | 13.9 (13.5–14.3) | 12.6 (12.3–12.9) | 9.1 (8.8–9.5) | 8.6 (8.1–9) |
|  | Standardized (95% CI) | 13.9 (13.5–14.3) | 12.5 (12.2–12.8) | 9.1 (8.8–9.4) | 8.6 (8.1–9) |
| Marijuana | Crude (95% CI) | 8.9 (8.6–9.3) | 8.5 (8.3–8.8) | 6.6 (6.3–6.9) | 6 (5.6–6.4) |
|  | Standardized (95% CI) | 8.9 (8.6–9.2) | 8.5 (8.2–8.7) | 6.6 (6.3–6.9) | 6.0 (5.6–6.4) |
| Inhalants | Crude (95% CI) | 12.3 (11.9–12.7) | 11.1 (10.8–11.4) | 7.5 (7.2–7.8) | 7 (6.5–7.4) |
|  | Standardized (95% CI) | 12.3 (11.9–12.7) | 11 (10.7–11.3) | 7.5 (7.2–7.8) | 7 (6.6–7.4) |
| Methamphetamine | Crude (95% CI) | 9.2 (8.9–9.6) | 8.6 (8.4–8.9) | 6.2 (5.9–6.5) | 5.7 (5.3–6) |
|  | Standardized (95% CI) | 9.2 (8.9–9.5) | 8.6 (8.3–8.8) | 6.2 (6–6.5) | 5.7 (5.3–6.1) |
| NPS | Crude (95% CI) | 9.5 (9.1–9.8) | 8.5 (8.2–8.7) | 5.6 (5.3–5.8) | 5.3 (4.9–5.7) |
|  | Standardized (95% CI) | 9.4 (9.1–9.8) | 8.4 (8.1–8.7) | 5.6 (5.3–5.8) | 5.3 (4.9–5.7) |
| Invited to use Controlled drugs |  |  |  |  |  |
| Any Controlled Drugs | Crude (95% CI) | 0.5 (0.4–0.6) | 0.5 (0.5–0.6) | 0.9 (0.8–1) | 0.9 (0.7–1) |
|  | Standardized (95% CI) | 0.5 (0.4–0.6) | 0.5 (0.4–0.6) | 0.9 (0.8–1) | 0.9 (0.7–1) |
| Marijuana | Crude (95% CI) | 0.3 (0.2–0.3) | 0.3 (0.3–0.4) | 0.7 (0.6–0.8) | 0.7 (0.6–0.8) |
|  | Standardized (95% CI) | 0.3 (0.2–0.3) | 0.3 (0.3–0.4) | 0.7 (0.6–0.8) | 0.7 (0.6–0.8) |
| Inhalants | Crude (95% CI) | 0.3 (0.3–0.4) | 0.3 (0.3–0.4) | 0.6 (0.5–0.7) | 0.6 (0.5–0.7) |
|  | Standardized (95% CI) | 0.3 (0.3–0.4) | 0.3 (0.3–0.4) | 0.6 (0.5–0.7) | 0.6 (0.5–0.8) |
| Methamphetamine | Crude (95% CI) | 0.3 (0.2–0.3) | 0.3 (0.3–0.4) | 0.7 (0.6–0.7) | 0.7 (0.5–0.8) |
|  | Standardized (95% CI) | 0.3 (0.2–0.3) | 0.3 (0.3–0.4) | 0.7 (0.6–0.7) | 0.7 (0.5–0.8) |
| NPS | Crude (95% CI) | 0.3 (0.2–0.3) | 0.3 (0.2–0.3) | 0.6 (0.5–0.7) | 0.6 (0.5–0.7) |
|  | Standardized (95% CI) | 0.3 (0.2–0.3) | 0.3 (0.2–0.3) | 0.6 (0.5–0.7) | 0.6 (0.5–0.7) |
| Irregular or Late Wake-up Time | Crude (95% CI) | 16.9 (16.5–17.3) | 17.5 (17.2–17.9) | 18.1 (17.7–18.6) | 17.3 (16.8–17.9) |
|  | Standardized (95% CI) | 16.9 (16.5–17.4) | 17.4 (17.1–17.8) | 18.2 (17.7–18.6) | 17.3 (16.8–17.9) |
| Irregular or Late Bedtime | Crude (95% CI) | 40.6 (40–41.2) | 41 (40.5–41.4) | 38.9 (38.4–39.5) | 38.6 (37.8–39.3) |
|  | Standardized (95% CI) | 40.5 (40–41.1) | 40.8 (40.3–41.2) | 38.7 (38.2–39.3) | 38.6 (37.8–39.3) |
| Skipping Breakfast Frequently | Crude (95% CI) | 3.7 (3.4–4) | 4.1 (3.9–4.3) | 4.8 (4.5–5) | 4.9 (4.6–5.3) |
|  | Standardized (95% CI) | 3.7 (3.4–3.9) | 4.0 (3.9–4.2) | 4.7 (4.5–5) | 5.0 (4.7–5.3) |
| Spending Alone Time Without Adult Supervision | Crude (95% CI) | 11.3 (10.9–11.6) | 12.8 (12.5–13.1) | 13.6 (13.2–14) | 11.5 (11–11.9) |
|  | Standardized (95% CI) | 11.2 (10.8–11.5) | 12.7 (12.4–13) | 13.5 (13.1–13.9) | 11.5 (11–12) |
| Dissatisfaction with School Life | Crude (95% CI) | 10.8 (10.4–11.1) | 10.4 (10.2–10.7) | 10.3 (10–10.7) | 9.3 (8.8–9.7) |
|  | Standardized (95% CI) | 10.8 (10.4–11.1) | 10.4 (10.1–10.7) | 10.3 (10–10.6) | 9.3 (8.8–9.7) |
| Lack of close friends | Crude (95% CI) | 3.4 (3.2–3.7) | 3.1 (2.9–3.3) | 3.2 (3–3.4) | 2.8 (2.6–3.1) |
|  | Standardized (95% CI) | 3.4 (3.2–3.7) | 3.1 (2.9–3.2) | 3.2 (3–3.4) | 2.8 (2.6–3) |
| Lacks friends to confide in | Crude (95% CI) | 10.2 (9.9–10.6) | 9.2 (9–9.5) | 10.6 (10.2–10.9) | 9.4 (9–9.8) |
|  | Standardized (95% CI) | 10.3 (9.9–10.7) | 9.2 (9–9.5) | 10.6 (10.2–10.9) | 9.4 (8.9–9.8) |
| Rarely consults with parents | Crude (95% CI) | 49 (48.5–49.6) | 46.6 (46.2–47.1) | 45.3 (44.7–45.9) | 43.5 (42.7–44.2) |
|  | Standardized (95% CI) | 49.2 (48.7–49.8) | 46.4 (46–46.9) | 45.3 (44.7–45.8) | 43.5 (42.7–44.2) |

Abbreviations: 95% CI, 95% confidence interval; NPS, novel psychoactive substance.

Prevalence estimates were presented as both crude and standardized percentages, with the 2024 survey population used as the reference for standardization. Standardized estimates were adjusted for sex and grade.

| **Variable Combination** | **Drug Use Proportion (%)** | **n (Drug Use)** | **n (Total)** |
| --- | --- | --- | --- |
| None of the three factors | 0.25 | 267 | 105,359 |
| Only Dissatisfaction with school life | 0.45 | 34 | 7639 |
| Only Lack of close friends | 0.84 | 13 | 1540 |
| Only Rarely consults with parents | 0.43 | 354 | 83021 |
| Dissatisfaction with school life × Lack of close friends | 0.47 | 5 | 1063 |
| Dissatisfaction with school life × Rarely consults with parents | 0.63 | 72 | 11422 |
| Lack of close friends × Rarely consults with parents | 0.99 | 19 | 1910 |
| All three factors | 3.34 | 68 | 2036 |

**Supplementary Table 3. Proportion of Lifetime Controlled Drug Use by Combinations of Social Risk Factors Among Junior High School Students**

Students were categorized based on the presence of the following three social risk factors: dissatisfaction with school life, lack of close friends, and rare consultations with parents.

The row “All three factors” refers to students who met all three criteria simultaneously.

**Notes:**
Drug Use Proportion (%) indicates the percentage of students in each group who reported lifetime use of any controlled substance.

Drug Use (n) refers to the number of students who reported drug use.

Where n (total) is the total number of students in each corresponding risk category.

For descriptive purposes, all values are based on unweighted counts.
